# 3D Cardiac microvessels embolization imaging based on X-ray phase contrast imaging

**DOI:** 10.1186/1475-925X-13-62

**Published:** 2014-05-21

**Authors:** Lu Zhang, Ke Wang, Fei Zheng, Xia Li, Shuqian Luo

**Affiliations:** 1 School of Biomedical engineering, Capital Medical University, Beijing, P.R. China; 2 Department of Physiology and Pathophysiology, School of Basic Medical Sciences, Capital Medical University, Beijing, P.R. China; 3 Beijing Friendship Hospital, Capital Medical University, Beijing, P.R. China

**Keywords:** X-ray phase contrast imaging (XPCI), Microvessel embolization imaging, Myocardial infarction (MI)

## Abstract

**Background:**

The treatment of microcirculatory impairment will have great impact if it can be applied to myocardial infarction (MI) patients. The problem is how to study these tiny structures and microphenomenon in heart.

**Methods:**

We investigated the visualization of cardiac microvessels embolization by the mean of X-ray phase contrast imaging (XPCI), which is a recently emerged imaging technique. Using the information of X-ray phase shift, it is sensitive to weak absorbing materials. Two MI SD rats were used as the microvessel embolization samples. MI was surgically induced by ligating left anterior descending artery. Imaging was performed 24 hours post-infarct, with barium sulfate as contrast agent.

**Results:**

The coronary arteries were visualized with smooth walls and clear edges. The ligated vessels, with the diameter of about three hundred microns, can be clearly distinguished and there were no distal blood flow downstream from these branches. The results indicate that phase contrast imaging can directly demonstrate the distribution of microvessels, and estimate the area of MI. The infarct location was in good agreement with pathological analyses of the models.

**Conclusions:**

The advantage of our method is directly observing and evaluating microvessel embolization which simplifies the procedure of diagnoses. Moreover, it is helpful for predicting the prognosis in MI and judging if angiogenesis happens.

## Background

The occlusion of coronary artery is the most common cause of Myocardial Infarction (MI) [1]. When MI happens, diminished blood supply of one or some coronary arteries will lead to myocardial ischemia, which will result in myocardial cell damage or death. To date, MI is still a worldwide disease with high morbidity and mortality. The main reason of that might be because of the link between the occurrence of MI and hyperlipidemia, diabetes mellitus, hypertension, smoking, obesity, lack of exercise and job stress. People, more or less, suffer from these risk factors [2].

Clinically, reperfusion has proven to be the vital procedure to improve the survival rate of MI patients. Recent studies have shown that even though after a rapid reopening of the previously obstructed coronary artery, some distal microvessels remain ischemia, which is called no-reflow phenomenon [3,4]. This abstruse, but important phenomenon is frequently happened during reperfusion of myocardial infarction. It has close relationship with patients’ chest pain, hemodynamic deterioration, ST-segment elevation, infarct extensions, ventricular arrhythmias, early congestive heart failure, and even cardiac rupture [5,6]. Therefore, the reperfusion of microvessels is currently of significant interest. The treatment of microcirculatory impairment will have greater impact if it can be applied to MI patients. The problem is how to study these tiny structures and microphenomenon in heart.

With the development of imaging technique, contrast echocardiography (MCE), positron emission tomography (PET), and contrast-enhanced magnetic resonance imaging (MRI) are becoming the most effective method for microvascular assessment. The area of ischemia can be defined through those methods. But the use of these techniques have been limited on imaging individual microvessels [7-9]. For the study of myocardial microcirculation, structure and function have the same importance. However, there are no ideal methods to realize cardiac microvessel imaging without destroying the original complete heart. Conventional histological studies only provide an accurate view of microvessels on cross-sectional specimens in two-dimension (2D). However, in order to study the cardiac microvessels embolization, three-dimemsional (3D) morphology of the blood vessel system has to be precisely determined. The 3D reconstruction of vascular network via a serious of sectioning is very complicated and time-consuming. Confocal laser scanning microscope (CLSM) has a high imaging resolution as well as 3D imaging ability. Unfortunately, the sample size should be very small and thin [10]. It is not suitable for a whole rat heart imaging.

X-ray Phase contrast imaing (XPCI) became a research focus about some decades ago with the awareness of its high spatial resolution and contrast on imaging soft tissues [11,12]. It is capable for detecting the direction deviation when X-rays travel through one object. Compared with clinical used X-ray mechanism, which relies on beam’s attenuation, XPCI is more sensitive to density variations in the sample. The X-rays direction change at the boundaries between different tissues, e.g. the interface between vessels and surrounding tissues, will be transformed into intensity change in the final image. The image contrast and resolution have been greatly improved. This superiority has been exploited for imaging malignant tumor, joint, cerebral vessels, lung airways, and etc. [13-16].

In order to observe blood vessels, contrast agents are widely used in conventional X-ray examination, including Digital subtraction angiography (DSA), Computed tomography angiography (CTA) and enhanced CT scanning. Though PCI can provide high contrast of soft tissues, we used contrast agent in our experiments to highlight microvessels in rat hearts. In this way, the image will compose more details of vessel networks due to phase contrast. Moreover, other soft tissues such us cardiac valves and myocardium can also be detected in the same image.

Accordingly, in this paper, we sort to directly visualize coronary microvessels, micro embolization and evaluate myocardial ischemia area by means of phase contrast imaging.

## Methods

### Myocardial infarction model

All experiments and procedures carried out on the animals were approved by the animal welfare committee of Capital Medical University and the approval ID is 0000259. Two adult normal SD rats, approximately 250 g of body weight each, were anesthetized by an intraperitoneal injection of chloral hydrate 4.5 ml/kg, tracheal intubated and connected to a small animal ventilator. MI was induced by ligating of the left anterior descending (LAD) coronary artery with a 6 absorbable suture through the left fourth intercostal space. Then we closed the thoracic cavity layer by layer and removed the endotracheal tube.

In order to observe the micro vascular network, contrast agent was used. But, in our experiment, the particles of contrast agent should be smaller than the capillaries. At the same time, the agent cannot migrate through the walls of the vessels. Moreover, it should be insoluble even though the tissue was fixed in formalin. After a serious of test, barium sulfate is proven to be the desirable one. To ensure microvessels angiography, it should be ground into very fine particles prior to use.

24 h after the operation, we anaesthetized the rats again, opened their thoracic cavity to expose the heart, cut the postcava, ligated the ascending aorta, and then injected the barium sulfate solution (20% v/v) by a scalp acupuncture, which connected to a 20 ml syringe, via the aorta root. After the coronary artery turned white, we harvested the hearts and quickly preserved them in formalin solution. Finally, phase contrast imaging was performed on these isolated hearts. After imaging, every heart was equally dissected into four blocks along the long axis of heart and paraffin embedded. 5 μm thick sections were cut and stained with hematoxylin and eosin (H&E).

### X-ray Phase contrast imaging

The coronary microvessels were visualized by X-ray phase contrast imaging. The principle of XPCI is Fresnel diffraction theory [17]. Contrast and resolution are derived from direct Fresnel diffraction with synchrotron radiation which has good spatial coherence and high intensity. When the beam traverse the object, X-ray undergo amplitude attenuation in addition to phase-shifts. After the downstream beam propagates for a sufficient distance, the phase-shifts are transformed into measurable intensity variations by means of Fresnel diffraction. Since the phase shift between biological soft tissues is almost one thousand times greater than the absorption term at photon energies greater than 10 keV, this technique can greatly improve the image quality of soft tissues.

**Figure 1:**
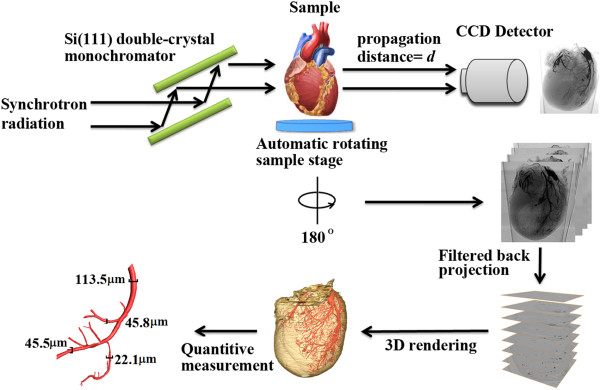
Schematic set-up of the XPCI system and the processing flow chart.

The imaging experiment was performed in Shanghai Synchrotron radiation facility BL13W beamline. Briefly, it consists of one double-crystal monochromator, one rotation sample stage and one CCD detector (Figure 1). The high-energy and high parallel synchrotron X-ray beam first cast on the monochromator crystals (Si 111 or Si 311) which can be rotated to select beam energy. The outcome beam became single energy X-ray. The tunable energy range was from 8 to 72.5 keV, with the energy resolution of about 0.5%. In our experiment, it was adjusted to 16.5 keV. This beam then travel through the imaging object. The direction associated with the intensity of the beam will change due to refraction, small angle scattering and attenuation of the beam. Subsequently, the transmitted beam propagated for some distance and then captured by the CCD detector. Just during this distance, the downstream beam was enhanced by Fresnel diffraction and the phase modulation was transformed into CCD receivable intensity.

For 2D imaging, the CCD detector we chosen had a resolution of 2048 × 2048 pixels with 3.7 μm × 3.7 μm each. The distance between sample and detector was 0.28 m. The explore time was 3.5 s. For CT experiment, the resolution of the detector was 4008 × 2672 pixels with 9 μm × 9 μm each. After the sample stage rotated for 180 degree, 1254 projection images were obtained, with an exposure time of 90 milliseconds each. The distance between sample and detector was 0.52 m. The surface dose was about 2.66 mGy for each projection. Filtered back projection method was used to reconstruct the cross-section of the sample [18]. The algorithm was implemented via Matlab 2009b (Mathworks Inc, Natick, MA, USA). Surface rendering later was used for 3D rendering of the heart by a 3D imaging software (Amira 5.2, Visage Imaging, Berlin, Germany).

## Results

**Figure 2:**
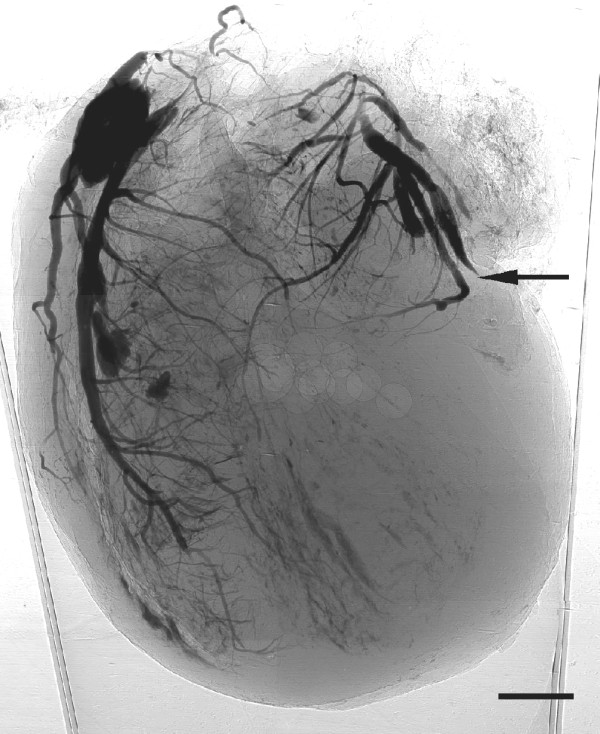
**Phase contrast image of the heart.** Pixel size = 3.7 μm. Scale bar = 1.5 mm.

**Figure 3:**
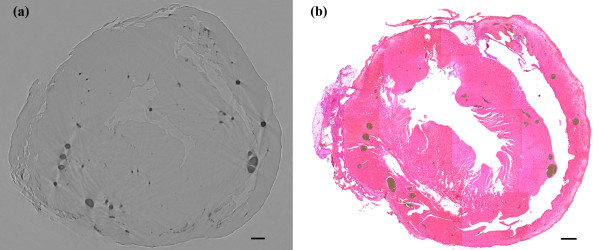
**Comparison of XPCI slice (a) and histological section (b).** Scale bar = 500 μm.

**Figure 4:**
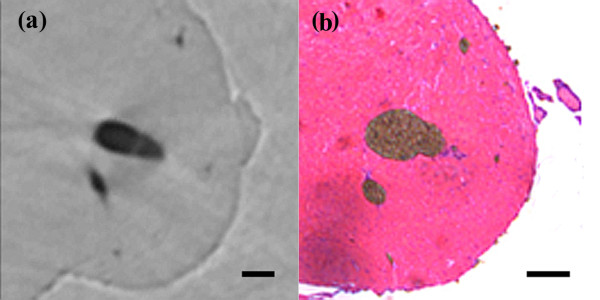
**Amplified microvessel region. (a)** XPCI image **(b)** Histological section. Scale bar = 100 μm.

The two rats were established as models of acute myocardial infarction. 24 h after infarction, we started the imaging test. The phase contrast image of the heart is shown in Figure 2. The vessels in heart were visualized with smooth walls and clear edges. The ligated vessels can be distinguished and no distal vessels of these branches as pointed by arrow. For the study of cardiac microvessels, depicting the 3D architecture of the vessels is real challenging, if we only imagine it from 2D projected images. In our study, we reconstructed the cross-section of the heart volume, shown in Figure 3. The result was in good agreement with pathological section. Since the vessels were filled with contrast agents, they turned dark brown color in pathological sections. For microvessels, we showed three vessels with diameter less than 100 μm in Figure 4. The corresponding section was found by tissue pathology with similar size and relative position.

**Figure 5:**
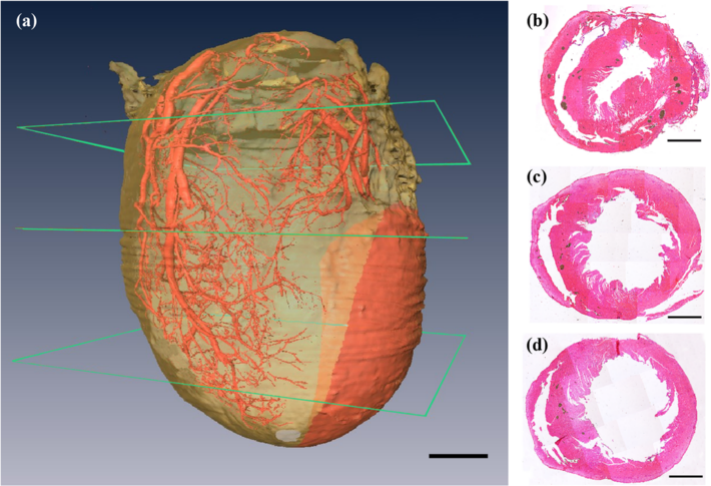
**3D cardiac vessel tree (a) and three corresponding histological slices are shown as (b), (c), (d) up to down separately.** The red area in **(a)** is the predicted myocardial infarction area. Scale bar = 2 mm.

**Figure 6:**
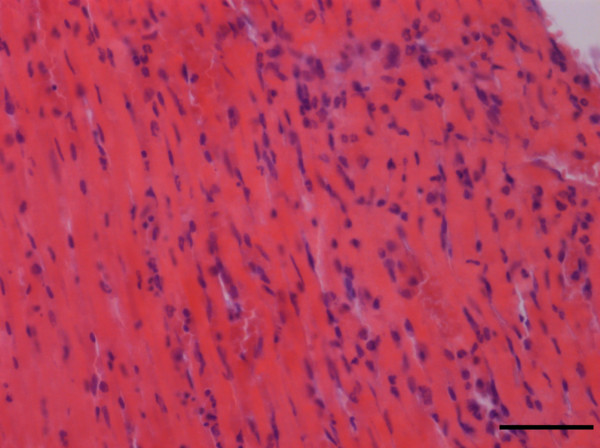
**Typical light microscopic feature of myocardial infarction with numerous neutrophils (H&E).** Scale bar = 50 μm.

Totally, we got 1677 phase contrast imaging slices. From the 3D rendered heart, the tree-like coronary arteries and the distal small arteries up to tenth level with a diameter of 15 μm were clearly visualized (Figure 5(a)). Since the ligated LAD coronary artery had no blood supply, the distal vessels were missing in the image. From this 3D model, we could directly find how many microvessels were blocked, where they were blocked and roughly identify where the heart muscle was not receiving enough blood supply (red part in Figure 5(a)). The use of histological stain is a widely accepted method to estimate the accuracy of phase contrast imaging. We equally divided the heart tissue into four blocks along the long axis of heart and then got three cross-sections. Three corresponding H&E staining histological slices were shown as (b), (c), (d) up to down separately. They were approximately the same as those part shown in Figure 5(a). The first cross-section was above the ligation position. Therefore, in this part, left and right coronary arteries existed in the image (Figure 5(b)). For the other two cross-sections, under the ligation position, left coronary artery was missing but the right was visible (Figure 5(c), (d)). The infarct area was also confirmed by H&E staining (Figure 6). The region had ischemic myocardium and with numerous neutrophils (Figure 6).

## Discussion

Our present study demonstrated that microvessel embolism is detectable and visible in myocardial infarction by phase contrast imaging. By assessing the no-blood-flow vessel, we have speculated the infarction area.

For myocardial infarction imaging, there are a couple of methods. CTA is widely used in clinic to evaluate coronary arteries diseases; DSA is currently considered as the diagnostic standard. Other modalities such as contrast enhanced MR, PET, and ultrasound are becoming the effective methods for infarction imaging [8,19,20]. In contrast to mark the ischemia region, direct visualization and quantification of the infarct vessel is more meaningful. In a number of clinical cases, after relief of the infracted large vessel, the blood flow of the ischemic tissue was still impeded which lead to poor prognosis. Till now there is no explicit explanation to this no-reflow phenomenon. Some researchers tend to believe that it is caused by microvessels obstruction [4,6]. For this reason, microvessel imaging in MI is gained more importance. Only a limited number of previous studies have mentioned cardiac microvessels imaging. Kiyooka et al. [21] observed coronary capillary by using their unique high-resolution intravital charge-coupled device video microscopy in two dimension (2D). However, one inherent limitation of 2D imaging is tissue overlap which make it difficult to distinguish spatial distribution of blood vessel. Eiji Toyota and associates [22] reported their work about 3D visualization of microvessels by synchrotron radiation micro-computed tomography. But they focused on the phasic change in resistance of microvessels during systole and diastole not the vessel visualization. Subsequently, Bert Müller et al. [23] used the same technique to image microvessels in some selected part. The thinnest vessel was on capillary level. However, the vessels appeared to be disconnected.

In the study of coronary heart disease, it is very important to directly observe the anatomic variations and anastomosis of microvessels. Thus, we imaged the whole rat hearts. Microvessels, such as coronary artery and some other arteries and veins were shown in our results. The infarct vessel can be directly found in the 3D vessel tree. For the part without vessel supply, we predict it as the ischemia area. The results were highly consistent with the histological section which is recognized as the accurate view of specimens.

The contrast agent we used in our experiment was barium sulfate. It can deposit in the vessel even after the heart is fixed in formalin solution. The histological result also proved this: the vessel lumen was completely filled with gray color contrast agent. The shortcoming is that the blood flow was solidified too fast in microvessels before barium sulfate contrast agent enter into them. The reason to choose barium sulfate lies in our in vitro heart imaging experiment. If we can image the heart in vivo, then clinical soluble contrast agent can be used. However, the most important problem to apply XPCI to in vivo heart imaging is the imaging speed. It is possible for 2D continuous heart imaging by XPCI. But for 3D imaging, the fast heart beating is still a big problem. Even some tiny movement will cause blur in 3D reconstruction of microvessels. In our opinion, the possible solution may be new high speed CCD and rotation stage.

## Conclusions

In conclusion, by using XPCI, it is possible to directly image microvessels in MI model and quantify the infarct microvessels. The quantitative character of the microvessels can be directly observed. The microvessel stenosis strongly correlates with clinical no-reflow phenomenon. Phase contrast imaging may become a new way to study MI. In the next step, we will explore the clinical significance of our findings, especially in no-reflow phenomenon.

## Competing interests

The authors declare that no competing interests exist.

## Authors’ contributions

LZ contributed to the XPCI data acquisition, image processing and manuscript drafting. KW and FZ established the MI rats model. XL is responsible for histological testing. SL participated in the design and supervised the experiment. All authors read and approved the final manuscript.
